# Long-term facial nerve function following facial reanimation after translabyrinthine vestibular schwannoma surgery: A comparison between sural grafting and VII–XII anastomosis

**DOI:** 10.3892/etm.2013.1120

**Published:** 2013-05-16

**Authors:** ZHAOYAN WANG, ZHIHUA ZHANG, QI HUANG, JUN YANG, HAO WU

**Affiliations:** 1 Department of Otolaryngology Head and Neck Surgery, Xinhua Hospital, Shanghai Jiaotong University, Shanghai 200092, P.R. China; 2 Ear Institute, Shanghai Jiaotong University, School of Medicine, Shanghai 200092, P.R. China

**Keywords:** facial reanimation, sural graft, VII-XII anastomosis, vestibular schwannoma

## Abstract

The aim of this study was to compare the recovery of long-term facial nerve function between patients who received sural grafts and those who underwent hypoglossal-facial anastomosis techniques following translabyrinthine vestibular schwannoma surgery. This study included 25 patients with vestibular schwannomas treated with translabyrinthine tumor removal. All patients had large tumors with a mean tumor size of 3.12 cm. Of these patients, six had progressive tumor enlargement symptoms and had been treated previously with stereotactic irradiation. Preoperatively, all patients had normal facial functions, and total tumor removal with a translabyrinthine approach was achieved in all cases. During surgery, the facial nerve was interrupted in all 25 patients. Two types of facial reanimation were performed. Sural grafts were placed in 13 patients and hypoglossal-facial (VII–XII) anastomosis was performed in the other 12. Facial nerve function and surgical outcomes were observed upon discharge, in the short term (one year following surgery), and in the long term (three years following surgery). Total facial paresis was observed in all patients upon discharge. In the sural graft group, House-Brackmann grade III facial function was achieved in four patients upon short-term evaluation and in ten upon long-term evaluation, while House-Brackmann grade IV facial function was achieved in nine patients upon short-term evaluation and three in the long term. In the VII-XII anastomosis group, House-Brackmann grade III facial function was achieved in two patients in the short term and eight in the long term, and House-Brackmann grade IV facial function was achieved in ten patients in the short term and four in the long term. There was a statistically significant difference in the facial recovery results between the short- and long-term follow-up periods. The sural graft group exhibited a marked improvement in results compared with the VII–XII anastomosis group, but no statistically significant difference in facial function was observed between the two facial reanimation groups at either the short- or long-term follow-up. In the sural graft group, synkinesia, noted in three patients, was the most frequently observed complication. Claudication was common upon discharge (four patients), but diminished during follow-up. Disarticulation was the most common complication in the VII–XII anastomosis group (five patients); numbness of the tongue was the second most common complication (four patients). None of the patients developed dysphagia. Facial reanimation is an effective procedure for the surgical rehabilitation of static and dynamic facial nerve functions. Significant improvement in facial nerve function may occur more than three years after surgery. Despite morbidities such as synkinesia, the sural graft technique demonstrates greater improvements in facial nerve function than VII–XII anastomosis in the short and long term following surgery, but this conclusion requires confirmation by larger studies with a greater number of patients.

## Introduction

Vestibular schwannoma surgery is currently performed with low morbidity and mortality rates due to improvements in diagnostic capabilities and advances in perioperative monitoring and microsurgical techniques (1). Intraoperative facial nerve interruption may be minimized, but remains an inevitable complication (2,3), particularly for patients with certain types of large tumors or recurrent tumors following stereotactic irradiation (4–7). The most common techniques for facial reanimation are sural grafting and hypoglossal-facial (VII–XII) anastomosis. It has been reported that these techniques produce excellent results, as assessed by the House-Brackmann grade of facial function, but to date there has been no long-term comparison of the techniques (6).

The aim of the current study was to compare the short-and long-term facial recovery results and surgical outcomes of patients who had undergone sural grafting and VII–XII anastomosis for the reanimation of interrupted facial nerves during the removal of translabyrinthine vestibular schwannomas.

## Patients and methods

### Subject selection

All vestibular schwannoma surgeries performed by surgeons at Xinhua Hospital (Shanghai, China) between 2003 and 2006 were screened for the following selection criteria: i) translabyrinthine total tumor removal; ii) normal preoperative facial nerve function (House-Brackmann grade); iii) interruption of the facial nerve during surgery and immediate reanimation by sural graft or VII–XII anastomosis; and iv) data available at all postoperative points in time (upon discharge from the hospital, at one year, and at three years following surgery). These criteria were met in 25 cases. These patients became the subjects of this study. Written informed consent was obtained from patents and their families. The study was approved by the Ethics Committee of Xinhua Hospital.

### Patient characteristics

The characteristics of the patients in the two groups are shown in Table I. There were no statistically significant differences in the mean age or tumor size.

The patients were divided into two groups, odd and even according to the last number of their ID. Odd patients received sural graft facial reanimation and even patients received VII–XII anastomosis. The four patients in whom it was not possible to identify the proximal stumps of the facial nerves were placed in the even group.

### Facial function and surgical data

Facial function and surgical data were obtained from Xinhua Hospital records. Patients who underwent vestibular schwannoma removal were routinely followed up for facial function and surgical outcome by interview approximately one and three years following surgery. All data were accessible for the purpose of this study.

### Follow-up

During the follow-up period, any complications developed by the facial reanimation patients, such as synkinesia, claudication, disarticulation, numbness of the tongue and dysphagia, were assessed.

### Statistical analysis

Statistical comparisons were performed using parametric and non-parametric tests, as appropriate. Assessments were performed using two-tailed probabilities. P<0.05 was considered to indicate a statistically significant difference. Statements showing no difference between groups indicate that a statistical test was performed and failed to reject the null hypothesis.

## Results

### Observations at discharge

**Figure 1.**
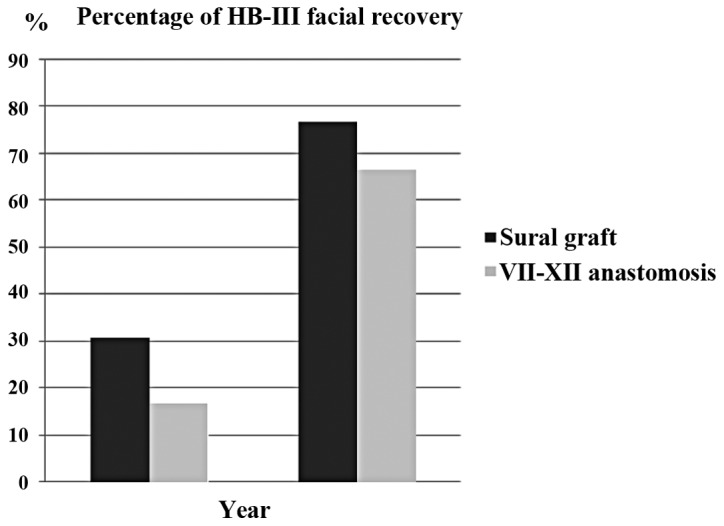
Sural graft patients exhibited a marked improvement in the recovery of facial nerve function during follow-up compared with VII–XII anastomosis patients, but there was no statistically significant difference across the treatment groups. Short term P=0.645; long term P=0.673.

House-Brackmann facial grade VI function was observed in all 25 patients upon discharge. Significant recovery of facial nerve function was observed at both postoperative time intervals: one year (short term) and three years (long term). Facial nerve grades for the two groups at the short- and long-term intervals are shown in Fig. 1 and Table II. There was a statistically significant difference in facial nerve recovery between the short- and long-term follow-up in the two treatment groups. In the sural graft group, House-Brackmann grade III facial function was achieved in four (30.8%) and ten (76.9%) patients in the short and long term, respectively, and House-Brackmann grade IV facial function was achieved in nine (69.2%) and three (23.1%) patients in the short and long term, respectively. There was a statistically significant difference between the facial recovery results across the short- and long-term follow-up periods in the sural graft group (P=0.036). In the VII-XII anastomosis group, House-Brackmann grade III facial function was achieved in two (16.7%) and eight (66.7%) patients in the short and long term, respectively and House-Brackmann grade IV facial function was achieved in ten (83.3%) patients in the short term and four (33.3%) patients in the long term. There was a statistically significant difference between the facial recovery results across the short- and long-term follow-up periods (P=0.047).

### Observations at follow-up

A comparison of the facial recovery results of the techniques revealed that, upon short-term follow-up, House-Brackmann grade III facial function was achieved in four (30.8%) and two (16.7%) cases in the sural graft and VII–XII anastomosis groups, respectively. Upon long-term follow-up, House-Brackmann grade III facial function was achieved in ten (76.9%) and eight (66.7%) cases in the sural graft and VII–XII anastomosis groups, respectively. It appeared that facial nerve function recovery was improved in the sural graft group compared with the VII–XII anastomosis group in both the short- and long-term follow-up, but no statistically significant difference was observed between the groups (short term, P=0.645; long term, P=0.673).

Some special outcomes following facial animation were observed during postoperative follow-up. In the sural graft group, claudication was common upon discharge (four patients), but diminished over time. Synkinesia, the most common cause of complaint, was observed in three patients. In the VII–XII anastomosis group, disarticulation was the most common complication, observed in five patients. Numbness of the tongue was the second most common complication, observed in four patients. None of the patients developed dysphagia and synkinesia.

## Discussion

In this type of large tumor, total tumor removal is achieved exclusively through the translabyrinthine approach (8,9). The translabyrinthine approach allows a positive identification of the distal segment of the facial nerve (10). Dissection of the plane between the tumor capsule and the facial nerve may then be extended medially (11). Following tumor debulking and the positive identification of the proximal facial nerve at the surface of the brain stem, the plane may be established medially. Combined medial and lateral identification provides the maximal possibility of preserving nerve integrity (12,13). It has been reported that particularly poor facial outcomes are observed in patients who required the surgical excision of previously irradiated vestibular schwannomas (14–16).

The incidence of facial nerve interruption has been reduced significantly due to improvements in surgical techniques and perioperative monitoring. It has been reported that automatic facial nerve preservation is achieved in >90% of large vestibular schwannoma patients. However, a certain degree of facial interruption is inevitable, particularly in patients with large or recurrent tumors (17).

Various techniques may be used for facial reanimation depending on the characteristic of the proximal stump of the facial nerve and the preference of the surgeon (18). Sural graft and VII–XII anastomosis are the most common facial reanimation techniques. Both surgeries provide the possibility of House-Brackmann grade III facial recovery in the long term. These techniques have advantages and disadvantages. The sural nerve has a history of use as a donor nerve, but there is evidence that motor nerve grafts are more appropriate than sensory nerve grafts (19). Acquisition of a sural cable may not necessarily extend the surgical period. With sural nerve grafting, the best outcome is House-Brackmann grade III facial function (20). Claudication is the most common complication at the time of discharge, but it quickly fades. Synkinesia is the most prominent complication of sural graft facial reanimation. VII–XII anastomosis may be achieved by a single surgical incision and does not require the identification of the proximal facial stump. The disadvantage of VII–XII anastomosis is that it sacrifices the function of the hypoglossal nerve (21). Disarticulation and numbness of the tongue are the most common complaints during follow-up. The best possible facial recovery is House-Brackmann grade III.

This study compared patients who underwent surgery during the same period by surgeons with the same level of experience. The mean patient age was 46.69 years in the sural graft group and 47.00 years in the VII–XII anastomosis group. No statistical difference in age was detected between the groups. Tumor size is the most important factor affecting facial function following surgery. The mean tumor size was 3.15 cm in the sural graft group and 3.08 cm in the VII–XII anastomosis group. No statistically significant difference was detected between the groups with regard to tumor size.

We compared the short- and long-term outcomes of facial reanimation. In the sural graft group, House-Brackmann grade III facial function was achieved in four patients in the short term and in ten patients in the long term. House-Brackmann grade IV facial function was achieved in nine patients in the short term and in three patients in the long term. In the VII–XII anastomosis group, House-Brackmann grade III facial function was achieved in two patients in the short term and eight patients in the long term. House-Brackmann grade IV facial function was achieved in ten patients in the short term and in four patients in the long term. There was a statistically significant difference between the facial recovery results across the short- and long-term follow-up periods. The outcomes of facial reanimation were acceptable following sural grafting and VII–XII anastomosis.

We compared the long-term outcomes of facial recovery following the different surgical facial reanimation techniques. Sural grafting appeared to produce superior facial recovery compared with VII–XII anastomosis in the short term (House-Brackmann grade III 30.8 vs. 16.7%) and in the long term (House-Brackmann grade III 76.9 vs. 66.7%). However, no statistically significant difference was observed between the surgical groups. Selecting the appropriate surgical technique for facial reanimation requires careful consideration of the complications and benefits of all available surgeries. A study sampling a greater number of patients may provide further insight into the value of various surgical procedures.

Following surgery, synkinesia developed in 23.1% (3/13) of sural graft cases in the present study. No significant synkinesia was observed in the VII–XII anastomosis group. This observation is explained by the fact that the healing of two nerves led to a greater incidence of incorrect regeneration by the facial nerve fibers (22).

Loss of function of the hypoglossal nerve led to numbness of the tongue and disarticulation, which was occasionally accompanied by severe facial paresis. Following surgery, rehabilitation training of the hypoglossal and facial nerves may improve functional recovery.

In conclusion, facial reanimation remains an effective procedure for the surgical rehabilitation of static and dynamic facial nerve functions. A significant improvement in facial nerve function may occur more than three years following surgery. Despite such morbidities as synkinesia, the sural graft technique is accompanied by a greater improvement in facial nerve function compared with VII–XII anastomosis in both the short- and long-term evaluations, but this requires confirmation by larger studies.

## Figures and Tables

**Table I.** Patient characteristics.

Variables	Surgical group		
	Sural graft (n=13)	VII–XII (n=12)	P-value
Age (years)			
Mean	46.69	47.00	0.934
SD	9.75	8.60	NS
Range	24–57	31–57	
Tumor size (cm)			
Mean	3.15	3.08	0.827
SD	0.88	0.70	NS
Range	2.0–5.0	2.0–4.5	

**Table II.** Comparison of short-term (1 year) and long-term (3 year) facial recovery.

HB	Sural graft		VII–XII anastomosis	
	1 year	3 years	1 year	3 years
Grade III	4	10	2	8
Grade IV	9	3	10	4
P-value	0.036		0.047	

HB, House-Brackmann.
